# Systemic inflammation response index predicts 3-month outcome in patients with mild acute ischemic stroke receiving intravenous thrombolysis

**DOI:** 10.3389/fneur.2023.1095668

**Published:** 2023-02-08

**Authors:** Min Chu, Yunhe Luo, Daosheng Wang, Yang Liu, Delong Wang, Yong Wang, Jing Zhao

**Affiliations:** ^1^Department of Neurology, Minhang Hospital, Fudan University, Shanghai, China; ^2^Department of Neurosurgery, Minhang Hospital, Fudan University, Shanghai, China

**Keywords:** systemic immune inflammation index, systemic inflammation response index, mild acute ischemic stroke, intravenous thrombolysis, 3-month outcome

## Abstract

**Introduction:**

A crucial aspect of stroke progression is the inflammatory response. As novel inflammatory and prognostic markers, the systemic immune inflammation index (SII) and the systemic inflammation response index (SIRI) have recently been studied. The objective of our study was to evaluate the prognostic value of SII and SIRI in mild acute ischemic stroke (AIS) patients following intravenous thrombolysis (IVT).

**Methods:**

Our study screened the clinical data of patients with mild AIS admitted to the Minhang Hospital of Fudan University for retrospective analysis. The SIRI and SII were examined by the emergency laboratory before IVT. Functional outcome was evaluated 3 months after the onset of stroke using the modified Rankin Scale (mRS). mRS ≥ 2 was defined as an unfavorable outcome. The relationship between SIRI and SII and the 3-month prognosis was determined using both univariate and multivariate analysis. Receiver operating characteristic curve was performed to evaluate the predictive value of SIRI for AIS prognosis.

**Results:**

A total of 240 patients were included in this study. Both SIRI and SII were higher in the unfavorable outcome group than in the favorable outcome group [1.28 (0.70–1.88) vs. 0.79 (0.51–1.08), *P* < 0.001 and 531.93 (377.55–797.12) vs. 397.23 (263.32–577.65), *P* < 0.001]. Multivariate logistic regression analyses showed that SIRI was significantly associated with 3-month unfavorable outcome of mild AIS patients [odds ratio (OR) = 2.938, 95% confidence interval (CI) = 1.805–4.782, *P* < 0.001], conversely, SII had no prognostic value. When SIRI combined with the established clinical factors, the area under the curve (AUC) showed a significant improvement (0.773 vs. 0.683, *P* for comparison = 0.0017).

**Conclusions:**

Higher SIRI could be valuable in predicting poor clinical outcomes for patients with mild AIS following IVT.

## Introduction

Worldwide, acute ischemic stroke (AIS) is a frequent condition characterized by high morbidity, disability, and mortality. Previous studies reported that more than half of AIS patients present with mild stroke (1, 2). Currently, there is no formal definition for mild AIS, but the majority of studies describe it as a National Institutes of Health stroke scale (NHISS) score ≤5 at admission (3, 4). In the clinical guidelines published in December 2019, intravenous thrombolysis (IVT) was recommended for mild AIS patients with disabling symptoms (5). However, ~30% of mild AIS patients have an unfavorable outcome after IVT (6, 7). Therefore, identification of risk factors linked to poor outcomes in patients with mild AIS after IVT is crucial. Age, diabetes, the baseline NHISS, and large vessel occlusion are some of the factors that have been mentioned in prior investigations (3, 8, 9). Moreover, it has been proposed that serum biomarkers may play an important role in the prognosis of mild AIS in recent years. Serum biomarkers, however, have received little research attention.

The blood-brain barrier disruption, oxidative stress, and the direct induction of neurocyte death that occurs as a result of inflammation response (IR) have all been identified as being important pathogenic processes of AIS (10, 11). Systemic immune inflammation index (SII) and systemic inflammation response index (SIRI), which are composed of platelets and three subtypes of white blood cells, have recently been reported as new inflammatory biomarkers that can reflect IR (12). Several studies have investigated the association between serum inflammatory indicators and functional outcomes in AIS patients (13–15). Additionally, it has been noted that the neutrophil-to-lymphocyte ratio (NLR) is a helpful inflammatory biomarker for predicting a bad short-term outcome in individuals with mild AIS following IVT, but their predictive effectiveness is not clarified (9).

Recently, SII and SIRI have been proven to predict the prognosis of some diseases such as ischemic stroke, coronary artery disease, subarachnoid hemorrhage and several cancers (16–19). However, the prognostic importance of SII and SIRI in mild AIS patients who have undergone thrombolysis has not yet been reported. Therefore, we aimed to systematically investigate the association of SII and SIRI with functional outcomes in mild AIS patients who received thrombolysis.

## Materials and methods

### Patients recruitment

This retrospective observational study was conducted from January 2017 to May 2022. All AIS patients receiving IVT therapy alone were consecutively included in the study and collected from the Minhang Hospital of Fudan University. An admission NHISS score ≤ 5 was considered to be mild AIS. The following were the inclusion requirements: (1) aged 18 years or older; (2) NHISS score ≤ 5 at admission; (3) stroke symptoms appearing within 4.5 h and receiving IVT treatment. Patients were disqualified if they fulfilled the following requirements: (1) score on the modified Rankin Scale (mRS) ≥ 2 before the stroke; (2) patients with malignant tumor, autoimmune disease and hepatic or renal diseases; (3) patients with acute infection, including pneumonia or other active concomitant infections. This study was reviewed and approved by the Ethical Review Board of Minhang Hospital of Fudan University.

### Data collection

Onset to treatment time (ONT), NHISS score and stroke subtype were evaluated by experienced clinicians. The Trial of Org 10172 in Acute Stroke Treatment (TOAST) criteria were used to classify stroke subtypes. Stroke nurses gathered demographic and baseline information on patients with age, gender, smoking, diabetes, hypertension, and atrial fibrillation. Before IVT, initial counts for platelets, monocytes, neutrophils, and white blood cells were also obtained. We calculated the SIRI and SII as follows: SIRI = NEUT × Mo/TLC, SII = PLT × NEUT/TLC, where PLT is the platelet count, NEUT is the neutrophil count, TLC is the total lymphocyte count and Mo is the monocyte count.

### Laboratory tests

Before thrombolysis, 2 mL of venous blood was collected with EDTA-K2 anticoagulant vacuum tube. Within 30 min after reversing evenly, Complete blood counts including white blood cells, neutrophils, lymphocytes, platelets, and other parameters were carried out using an automatic Mindray cal 8000 blood cell analyzer.

### Clinical outcome

Functional outcome was evaluated by mRS score at 3 months after the onset of stroke. There was a neurologist in charge of the follow-up using the phone and face-to-face follow-up. Favorable outcome was considered to be an mRS score ≤ 1, while an unfavorable outcome was considered an mRS score ≥ 2. Safety outcomes included hemorrhagic transformation (HT) and symptomatic intracerebral hemorrhage (sICH). HT was confirmed by computed tomography (CT) scan within 7 days after IVT therapy. Any intracranial hemorrhage that raises the overall NIHSS score by 4 points is considered to be a sICH.

### Imaging analysis

To determine the location of the infarction, all patients underwent brain magnetic resonance imaging (MRI). Ipsilateral severe vessel stenosis (ISVS) and ipsilateral severe vessel occlusion (ISVO) were evaluated by computed tomographic angiography (CTA). For patients with incomplete CTA, brain magnetic resonance angiography (MRA) examination was performed after admission. An intracranial or extracranial artery on the same side of the infarction with a diameter loss higher than 70% was categorized as ISVS. ISVO was defined as the absence of a blood flow signal from the ipsilateral infarction. Blinded to the clinical data, two seasoned MRI professional neuroradiologists separately assessed each subject's imaging manifestations, including the degree of artery stenosis and HT.

### Statistical analysis

Statistical analyses were performed using SPSS (version 26.0, IBM Corp, Armonk, NY, USA). Receiver operating characteristic (ROC) analysis was conducted to identify the optimal cutoff values of SII and SIRI for predictive clinical outcomes in mild AIS patients after IVT. All subjects were divided into two groups according to the mRS score at 3 months (favorable 0–1 vs. unfavorable 2–6). Normal-distribution continuous data were reported as mean ± SD and compared using the *t*-test, whereas non-normal-distribution continuous variables were expressed as median (interquartile range) and compared using the Mann–Whitney *U* test. Categorical variables were presented numerically (percentages, %) and compared between the groups using the relevant Fisher exact or Pearson χ^2^ tests. A subsequent multivariate logistic regression analysis included the variables for which *P* < 0.1 in the univariate analysis. *P* < 0.05 (two-sided) was used to determine statistical significance.

## Results

### Clinical characteristics of patients

**Figure 1:**
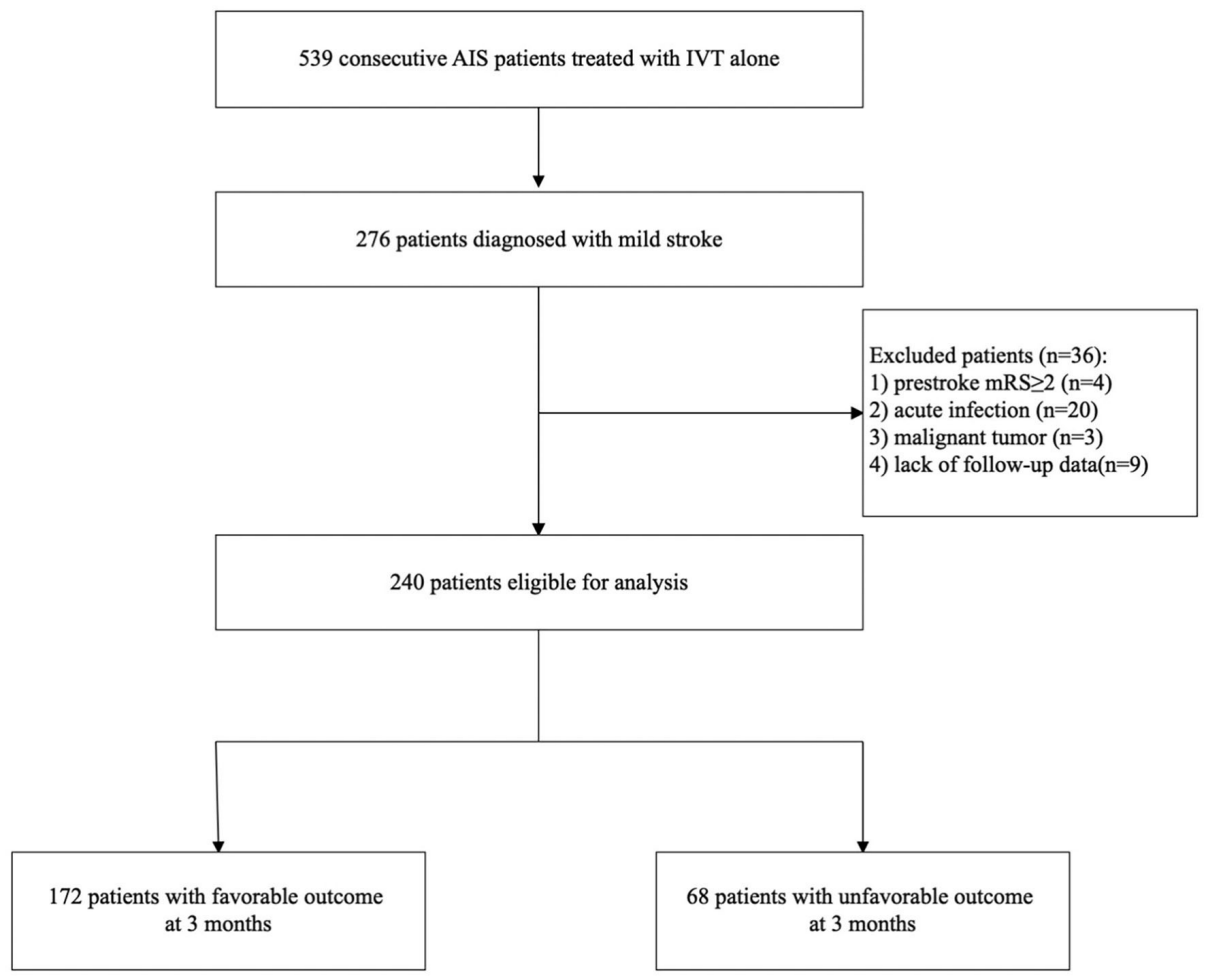
Diagram of the study recruitment.

During the study period, we collected 539 consecutive AIS patients treated with IVT alone. Of these patients, 276 patients were diagnosed with mild stroke. Finally, 240 patients were involved in our analysis after excluding those with pre-stroke mRS ≥ 2 (*n* = 4), acute infection (*n* = 20) including 18 patients with pneumonia and 2 patients with cholecystitis, malignant tumor (*n* = 3), lack of follow-up data (*n* = 9). Figure 1 shows a diagram depicting study recruitment.

There were 159 men and 81 women, aged 32–92 (median 66) years. Vascular risk factors included diabetes mellitus (*n* = 74, 30.8%), atrial fibrillation (*n* = 41, 17.1%), hypertension (*n* = 140, 58.3%), and smoking (*n* = 73, 30.4%). All patients were categorized into two subgroups according to the mRS score at 3 months. Compared with the patients in the favorable outcome group, the patients in the unfavorable group were older (66.00 vs. 65.00, *P* = 0.039). In terms of laboratory findings, the white blood cell level (7.42 vs. 6.51, *P* < 0.001), neutrophil count level (4.97 vs. 3.96, *P* < 0.001), monocyte count level (0.42 vs. 0.38, *P* < 0.001) were significantly increased in the unfavorable outcome group. The unfavorable outcome group had significantly higher SIRI (1.28 vs. 0.79, *P* < 0.001) and SII (531.93 vs. 397.23, *P* < 0.001) than the favorable outcome group and exhibited more serious neurological deficits on admission (NIHSS score 4.0 vs. 3.0, *P* = 0.002). The proportion of ISVS (25.0 vs. 13.4%, *P* = 0.029), ISVO (11.8 vs. 4.7%, *P* = 0.047), and ISVS/ISVO (36.8 vs. 18.0%, *P* = 0.002) in unfavorable outcome group were higher than in the favorable outcome group. Demographic features and risk factors are summarized in Table 1.

**Table 1:** Baseline characteristics of mild AIS stroke patients receiving IVT therapy based on favorable vs. unfavorable outcome at 3 months.

**Variables**	**All patients (*n* = 240)**	**Favorable outcome (*n* = 178)**	**Unfavorable outcome (*n* = 62)**	**P-value**
**Demographics**				
Age (year)	66.00 (60.00–73.35)	65.00 (58.35–72.00)	68.00 (62.00–75.75)	0.039^*^
Men, *n* (%)	81 (33.8%)	60 (34.9%)	21 (30.9%)	0.555
**Medical history**				
Hypertension, *n* (%)	140 (58.3%)	99 (57.6%)	41 (60.3%)	0.698
Diabetes mellitus, *n* (%)	74 (30.8%)	51 (29.7%)	23 (33.8%)	0.528
Atrial fibrillation, *n* (%)	41 (17.1%)	31 (18.0%)	10 (14.7%)	0.538
Current smoking, *n* (%)	73 (30.4%)	49 (28.5%)	24 (35.3%)	0.302
OTT (min)	145.00 (110.25–182.25)	143.00 (110.00–179.50)	155.00 (116.25–195.75)	0.143
NHISS score at admission	3.00 (2.00–4.00)	3.00 (2.00–4.00)	4.00 (3.00–4.75)	0.002^*^
**Stroke subtype**, ***n*** **(%)**				0.410
LAA	86 (35.8%)	56 (32.6%)	29 (42.6%)	
CE	35 (14.6%)	26 (15.1%)	9 (13.2%)	
SAO	89 (37.1%)	64 (37.2%)	25 (36.8%)	
SAE	3 (1.3%)	3 (1.7%)	1 (1.5%)	
SUE	27 (11.3%)	23 (13.4%)	4 (5.9%)	
HT, *n* (%)	20 (8.3%)	12 (7.0%)	8 (11.8%)	0.227
sICH, *n* (%)	4 (1.7%)	2 (1.2%)	2 (2.9%)	0.332
**Vascular stenosis**, ***n*** **(%)**				
ISVS	40 (16.7%)	23 (13.4%)	17 (25.0%)	0.029^*^
ISVO	16 (6.7%)	8 (4.7%)	8 (11.8%)	0.047^*^
ISVS/ISVO	56 (23.3%)	31 (18.0%)	25 (36.8%)	0.002^*^
**Laboratory measures**				
WBC counts (10^9^/L)	6.95 (5.67–8.10)	6.51 (5.45–7.95)	7.42 (6.62–8.75)	<0.001^*^
Neutrophil counts (10^9^/L)	4.18 (3.10–5.37)	3.93 (2.98–4.86)	5.11 (4.08–6.17)	<0.001^*^
Lymphocyte counts (10^9^/L)	1.90 (1.40–2.51)	1.97 (1.48–2.51)	1.77 (1.27–2.54)	0.195
Monocyte counts (10^9^/L)	0.39 (0.32–0.50)	0.38 (0.31–0.47)	0.42 (0.36–0.58)	<0.001^*^
Platelet counts (10^9^/L)	197.50 (164.24–236.75)	194.00 (159.50–237.25)	202.50 (174.50–236.75)	0.443
Hemoglobin (g/L)	142.00 (132.00–153.00)	142.00 (133.00–150.75)	140.00 (127.50–156.75)	0.800
SIRI	0.90 (0.55–1.34)	0.79 (0.51–1.08)	1.28 (0.70–1.88)	<0.001^*^
SII	434.51 (285.30–650.60)	397.23 (263.32–577.65)	531.93 (377.55–797.12)	<0.001^*^

* Means P < 0.05.

LAA, large-artery atherosclerosis; CE, cardioembolism; SAO, small-artery occlusion; SOE, stroke of other determined etiology; SOE, stroke of undetermined etiology; ISVS, ipsilateral severe vessel stenosis; ISVO, ipsilateral severe vessel occlusion; HT, hemorrhagic transformation; sICH, symptomatic intracerebral hemorrhage; SIRI, systemic inflammation response index; SII, systemic immune-inflammation index.

### Multivariate analysis of factors related to unfavorable outcome

In mild AIS patients who were only receiving IVT, univariate and multivariate logistic regression analyses were run to assess the prognostic significance of SIRI and SII. We included age, NHISS score at admission, diabetes mellitus, ISVS/ISVO, SIRI and SII into the multivariate analysis.

In multivariate analysis, the NHISS score at admission [OR = 1.430, 95% confidence interval (CI) = 1.114–1.835, *P* = 0.005], ISVS/ISVO (OR = 2.347, 95% CI = 1.173–4.696, *P* = 0.016), and SIRI (OR = 2.938, 95% CI = 1.805–4.782, *P* < 0.001) were the predictors of an unfavorable outcome (Table 2).

**Table 2:** Multivariate regression analysis of factors related to 3-month unfavorable outcome.

**Variables**	**Univariate**		**Multivariate**	
	**OR (95% CI)**	**P-value**	**OR (95% CI)**	**P-value**
Age (year)	1.029 (1.004–1.055)	0.024	–	0.119
NHISS score at admission	1.424 (1.130–1.795)	0.003	1.430 (1.114–1.835)	0.005
Diabetes mellitus	0.825 (0.453–1.502)	0.529	–	0.977
ISVS/ISVO	2.644 (1.411–4.954)	0.002	2.347 (1.173–4.696)	0.016
SIRI	3.101 (1.918–5.015)	<0.001	2.938 (1.805–4.782)	<0.001
SII	1.002 (1.001–1.003)	0.001	–	0.918

### Association of SIRI with clinical outcome

**Figure 2:**
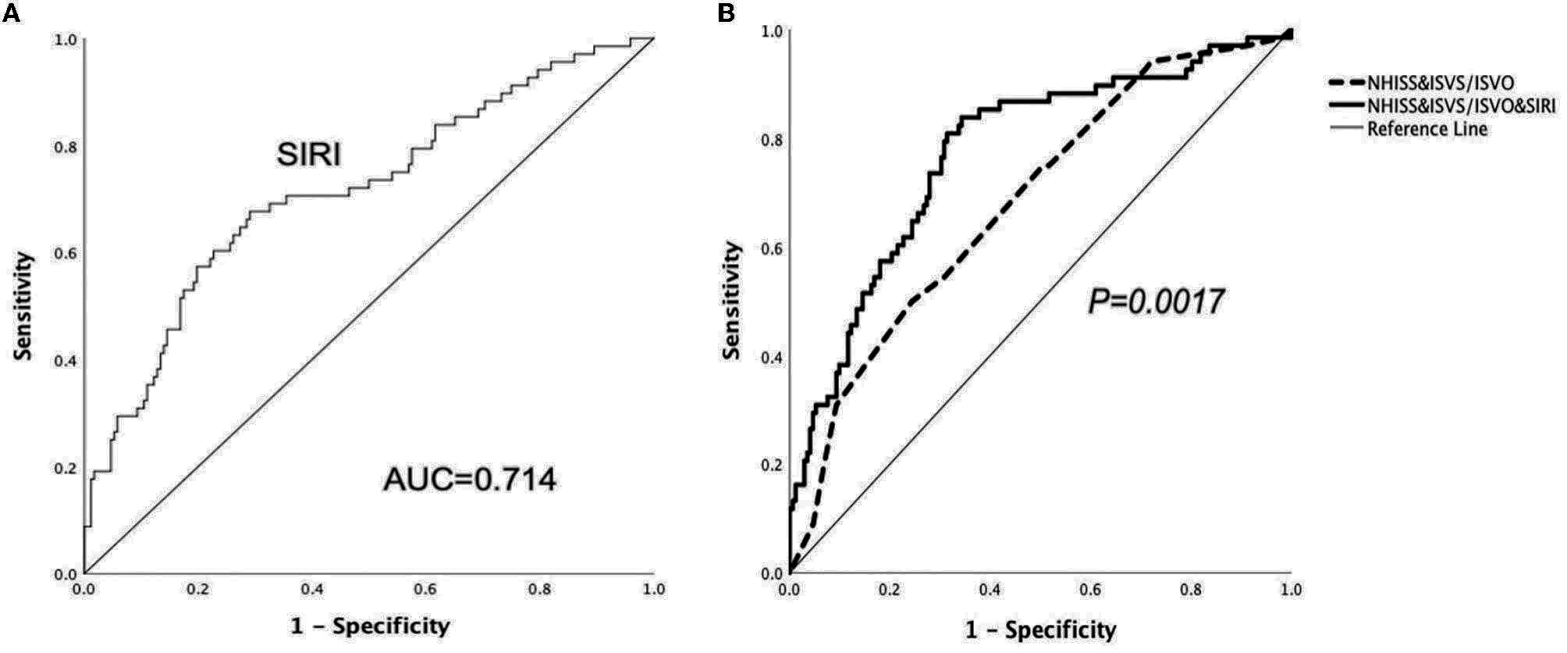
ROC curves demonstrate the predictive ability of SIRI and show SIRI improves the predictive ability of ISVS/ISVO&NHISS for unfavorable outcomes in mild AIS.

According to the ROC analysis (Figure 2), the optimal cut-off threshold of SIRI was 1.00 × 10^9^/L [area under the curve (AUC) = 0.714, 95% CI = 0.652–0.770, *P* < 0.001]. The ROC curve showed that the AUC of NHISS score combined with ISVS/ISVO to predict the adverse outcome after thrombolysis in mild stroke was 0.683 (95% CI = 0.620–0.742, *P* < 0.001). When SIRI combined the above indicators, the AUC increased to 0.773 (95% CI = 0.715–0.825, *P* < 0.001). There was a significant statistical difference between the two ROC curves (*P* = 0.0017).

## Discussion

For the first time, the current study examined the relationship between SIRI and SII and the 3-month functional outcome of mild AIS after IVT therapy alone. Our analysis demonstrated that SIRI and SII in patients with 3-month unfavorable outcomes of mild AIS treated with IVT are higher than those in patients with favorable outcomes. SIRI was an independent predictor for unfavorable outcome. Most importantly, when SIRI was combined, it can enhance the prognostic usefulness of traditional risk variables in people who had mild stroke after IVT.

It has been proved that the IR after AIS is related to the secondary brain damage after infarction (20, 21). The inflammatory mediators, cytokines, adhesion molecules, and chemokines released by immune inflammatory cells exacerbate tissue damage. According to previous studies, IR can be activated immediately after stroke event has occurred and is associated with poor prognosis (22–24). The mentioned mechanisms explain why the biomarkers now being researched are based on numerous inflammatory variables linked to stroke.

SIRI is a novel inflammation index, including peripheral neutrophils, lymphocytes and monocytes. Peripheral circulating neutrophils are regarded as the first inflammatory cells to penetrate the ischemic parenchyma after the onset of AIS, which exacerbate brain injury by releasing particles containing antibacterial enzymes and chemicals (25, 26). Higher neutrophil levels in early AIS were associated with larger infarction size. This may be because the increase in neutrophil concentration will promote the enhanced expression of matrix metalloproteinase-9 (MMP-9), a protein related to blood brain barrier damage (27, 28). In addition, monocytes are another important type of inflammatory cell after AIS, which can infiltrate infarcted areas and aggravate brain injury (29–31). Nevertheless, in contrast to neutrophils and monocytes, certain lymphocytes largely serve a protective role in the IR following AIS, controlling and reducing local IR (32). As a result, high SIRI (an increase in neutrophils, monocytes, and a decrease in lymphocytes) may accurately reflect adaptive immune response and IR, which are crucial processes for the incidence of stroke and may be effective biomarkers for prognosis.

Several earlier research has shown that SIRI is an effective marker for assessing the prognosis of varieties of inflammation-related diseases, which was consistent with our research results. According to Qi et al. (33), SIRI can be used to evaluate survival in pancreatic cancer patients receiving chemotherapy. Recently, Yun et al. (34) analyzed 680 aneurysmal subarachnoid hemorrhage (aSAH) patients and concluded that SIRI is an independent risk factor for unfavorable outcomes in aSAH patients. In addition, a retrospective study found that SIRI may serve as a predictive index for patients undergoing mechanical thrombectomy (MT) due to large artery occlusion (12). However, no study has found an association between SIRI and mild AIS treated with IVT. To the best of our knowledge, this is the first study to show a potential correlation between SIRI and the outcome of mild AIS after IVT.

Inflammatory markers based on platelets as well as leukocyte-based markers have been investigated in AIS patients. During AIS, platelets aggregate rapidly after blood vessel damage and play an important role in thrombosis. Furthermore, platelets also participate in immune inflammatory reaction. By altering the surface expression of *P*-selectin, platelets can directly interact with circulating leukocytes to create platelet-leukocyte aggregates and activate innate immunity in ischemic organs (35). Recently, the prognosis of AIS patients is thought to be reflected by SII, which is based on a combination of platelets, neutrophils, and lymphocytes (13). Even though the SII level in the group with a poor prognosis was higher than that in the group with a good prognosis in our study of thrombolytic patients with mild stroke, we did not discover that the SII was directly associated with the unfavorable outcome at 3 months. As it might be a viable clinical tool for determining mild AIS and its potential consequences, a more thorough and well-designed study on large populations of patients is required.

It has been demonstrated that the NHISS score at admission is a reliable predictor of a poor outcome in moderate AIS following IVT (3, 6). Our findings are consistent with those of earlier research. Additionally, we found that ISVS/ISVO was a predictor of a worse clinical outcome at 3 months, which is similar to a finding from a prior study (9). It reflects the bad condition of intracranial vessels and extracranial vessels.

There are some strengths in our study. First off, to the best of our knowledge, it is the first article that has specifically addressed the correlation between the short-term unfavorable outcome in patients with mild AIS following IVT and novel inflammation index, including SIRI and SII. Second, our study has a benefit over many earlier studies in that we investigated the predictive potency for the prognosis of mild AIS. However, our study also has some limitations that need to be considered. First of all, this analysis was performed retrospectively and without blinding, which has a chance of selection bias. Second, the absence of dynamic SIRI and SII data prevented us from making predictions that might be more accurate. Third, given the limited sample size, the results should be confirmed in future, larger populations.

## Conclusion

In conclusion, the current investigation shows for the first time that mild AIS patients with poor outcomes after IVT had increased SIRI and SII. Also, SIRI was an independent predictor for unfavorable outcomes at 3 months. Our work provides an economically convenient way to refine risk stratification for adverse outcomes in mild AIS. Further large-scale prospective studies are needed for dynamically monitoring SIRI and SII to understand the value of the index better.

## Data availability statement

The original contributions presented in the study are included in the article/supplementary material, further inquiries can be directed to the corresponding author.

## Ethics statement

The studies involving human participants were reviewed and approved by Ethics Committee of Minhang Hospital of Fudan University. The patients/participants provided their written informed consent to participate in this study.

## Author contributions

MC and JZ designed the study. YaL and YW collected the data. MC, YuL, and DaW performed the statistical analysis and wrote the paper. DeW and JZ revised the paper. All authors contributed to the article and approved the submitted version.
